# Locating regional health policy: Institutions, politics, and practices

**DOI:** 10.1177/1468018115599819

**Published:** 2015-12

**Authors:** Pia Riggirozzi, Nicola Yeates

**Affiliations:** University of Southampton, UK; The Open University, UK

**Keywords:** Health diplomacy, global health governance, regional integration, SADC, UNASUR

## Abstract

Poverty reduction and health became central in the agendas of Southern regional organisations in the last two decades. Yet, little is known about how these organisations address poverty, inclusion and social inequality, and how Southern regional formations are engaging in power constellations, institutions, processes, interests and ideological positions within different spheres of governance. This article reviews academic literatures spanning global social policy, regional studies and diplomacy studies, and the state of knowledge and understanding of the ‘place’ of regional actors in health governance as a global political practice therein. It identifies theoretical and thematic points of connection between disparate literatures and how these can be bridged through research focusing on the social policies of regional organisations and regional integration processes. This framework hence locates the contributions of each of the research articles of this Special Issue of Global Social Policy on the regional dimension of health policy and diplomacy in relation to Southern Africa and South America. It also highlights the ways in which the articles bring new evidence about how social relations of welfare are being (re)made over larger scales and how regional actors may initiate new norms to improve health rights in international arenas engaging in new forms of ‘regional’ diplomacy.

## Introduction

The presence of world-regional actors in spheres and practices of public policy-making and governance is taking hold as a vibrant subject of research and political agendas focused on-going processes of restructuring of social policy-making and delivery. Multidisciplinary programmes of social research are developing alongside pluralistic programmes of political action in recognition of the significance of regionalisation processes and regionalist modes of institutional formation, organisation, governance, policy and action in the making of social policy at national, regional and global levels. Increasingly, regional governing bodies and regional networks of state and non-state actors internationally are engaging with social policy issues within a regional frame, not only in the context of the European Union but also across the Global South (Deacon et al., 2010; Munck and Hyland, 2014; Yeates and Deacon, 2010). Regional organisations are themselves becoming actors in the (re)making of global relations of power, inequality and development (Deacon et al., 2010; Riggirozzi, 2015; Söderbaum and Van Langenhove, 2006).

The analytical, political and practice contexts and antecedents of this regionalist resurgence are many. A major theme in global governance during the last two decades is the growth of pluralistic structures and actors and, with this, recognition of countervailing sources of power that, together, are challenging totalising accounts of the sources, processes and impacts of economic and political globalisations. As Yeates and Deacon have argued, political resistance to Northern-driven global social reform agendas that may sustain minimalistic social commitments, Northern protectionism and Southern dependency has increasingly pointed towards the possibilities of creating new countervailing and pluralistic sources of power and policy spaces better attuned to the developmental and self-determination interests of the Global South (Yeates and Deacon, 2010: 28). In this context, fostering and sustaining distinctive regional practices and new ambitions in support of more socially just forms of production and consumption becomes a strategic possibility. It is not just that free-floating globalisation ‘touches down’ in distinct ‘local’ contexts or that global governance impacts ‘locally’, but that globalisation is variously generated, sustained, governed and modified by the multitude of actors and strategies seeking to affect both North–South and South–South development agendas (Yeates, 1999, 2001, 2002) – and this includes regional actors, processes and ‘new’ spaces of governance. Loosening the straightjacket of the ideological competition that subsumed regions to a sphere of influence during the Cold War world, since the 1990s the renewed emphasis on what ‘new regionalism’ means as a site of policy activism and as a policy agenda in itself has become a subject of inquiry. In a period of rapid global transformations, regional organisations, actors, policies, identities and forms of cooperation are shaping spaces for thinking – and enacting – alternative models and strategies of political and social engagement in support of a more robust social policy agenda. These are intriguing entry-points for a substantive and dynamic research agenda into the role of regional organisations in areas going well beyond the traditional spheres of commercial trade and investment policies, and which explores what commitments are being set and implemented in other policy domains and policy processes, and the ways in which they may be challenging the normative parameters dominating the so-called new regionalism of the 1980s and 1990s.

Of course, principled arguments for a stronger social policy project embedded in international integration processes have been made for some time (Deacon et al., 2007, 2010; Yeates, 2014a, 2014b, 2014d; Yeates and Deacon, 2010). Greater cross-border cooperation in social policy is needed to compensate for the limitations of market policies and address cross-border social harms, not least those that are generated or exacerbated by greater international integration and globalisation processes. At one level, regionalism provides an additional integrative scale for thinking about – and enacting – these kinds of cross-border cooperation and collaboration. Some empirical research to date has offered prima facie studies on regional groupings in the Global South affecting the political and social foundations of activism in the area of health (Fourie, 2013; Riggirozzi, 2015), and in the pursuit of collective public goods, alongside a defence of standards of procedural democracy (Riggirozzi and Grugel, 2015) but not enough is yet known about how Southern regional formations’ *social* policy ambitions, mandates and practices are manifesting themselves in practice, whether regionalist politics and policy are conducive to the emergence and definition of new (and progressive) courses of action in support of alternative modalities of progressive social policy and governance – and if they are, what are the conditions that are enabling this.

The various contributions to this Special Issue on the regional politics and practices of governance and diplomacy propose an analysis that moves away from one-dimensional views positing regionalism as a defensive and reactive response to various imperatives emanating from the global economy and the Global North, to set out a complex picture emphasising the multifaceted, dynamic, context-specific and ultimately ‘socio-political’ dimensions and impacts of regionalism in which regionalism is a significant force in the (re)making of global relations of power, inequality and development. We argue that regional formations are playing a significant role in shaping the formation of new socio-political ‘intra-regional’ agendas, as well as the potential to engage as a global actor through ‘extra-regional’ diplomacy and ‘bloc activism’ in support of those agendas. Exploring processes of regionalisation of social goals and welfare policies both advances a greater understanding of formal and informal processes driving regionalism beyond market-led objectives and provides much-needed social policy–relevant insights substantiating a wider appreciation of the meaning of *regional* integration as a ‘living’ set of socio-political ideas, policies and practices (Yeates and Riggirozzi, 2015: 8, 18).

This Special Issue addresses regional governance, policy, activism and diplomacy specifically in relation to the institutional policy sphere of health. It is organised in two principal sections: the first section comprises five research articles examining different manifestations of contemporary world-regional health governance and diplomacy; the second section, Forum, incorporates six shorter policy-focused articles examining the ‘place’ and potential of regionalism in the context of the post-2015 development agenda and Sustainable Development Goals. The empirical focus of this collection lies with Southern regionalisms spanning Southern Africa, Central and South America, and South-East Asia and with the ways in which they are forging and exploiting strategic spaces in global and regional health agendas in the interests of health as a public good, the right to health and access to health and the conditions and means for doing so. We consider the place of Northern regionalisms in relation to southern ones, but as a contextual and comparative device.

The Special Issue is part of a wider research agenda being taken forward in the context of a major international research programme on world-regional social policy developments in Southern Africa and South America, funded by the UK Economic and Social Research Council/Department for International Development. The project, Poverty Reduction and Regional Integration (PRARI), places a specific emphasis on regional health policies and their capabilities to become significant platforms for pro-social equity policy agendas and the mobilisation of diverse social actors in the interests of social inclusivity (see http://www.open.ac.uk/socialsciences/prari/).

In this context, our present focus on Southern regional institutions as sites of policy-making and as international actors adds important analytical and geographical nuances to scholarly work spanning multiple fields of academic inquiry. Academic literatures in International Relations have mostly been confined to traditional foreign (economic, security) policy dynamics of regional formations in the Global North (in particular, the European Union) and have not examined how foreign policy or development objectives are pursued through social policy domains. Lapsing into neither romanticism nor defeatism, the articles in this collection demonstrate how Southern regionalism may constitute strategically significant spaces for the advancement of social policy agendas, intra-regionally and extra-regionally. Building on previous research, the collection provides important further evidence of how regional organisations are framing political agendas as to what the purpose of regional integration should be, what kinds of social policies are needed and over what ‘integrative scales’ these social policies should be developed (Yeates, 2014a: 147; see also Riggirozzi, 2014) – together with the extent of context-specificity in terms of how these political and policy frames emerge and develop. The articles collectively show the degree of variation with regard to the adoption and pursuit of regional policies; how these translate into tangible achievements in national settings; the conditions under which distinctive regional agendas emerge; and the diverse strategies, actors and the constellations of interests and resources mobilised, not least through their own activism and practices of ‘diplomacy’ shaping policy agendas in regional and global areas.

Global Social Policy literatures have long recognised the significance of sub-global (regional) structures, institutions, actors and policies in shaping the global and national politics of social policy and welfare (Deacon et al., 2010; Yeates, 2007, 2014a, 2014d; Yeates and Deacon, 2010). Deacon et al.’s 2010 study, for example, provided the first extensively international evidence base on the social policy dimensions of world-regional social policy. That study showed the extent of engagement by regional associations of nations across four continents with questions of the relationship between trade, labour and social standards, and of how to maintain fiscal capacity and social solidarity in the face of international competition, and the extent of social policy mandates, goals, strategies and programmes around issues of health, welfare, education, food and wider socio-economic security, and social development (see also Yeates and Deacon, 2010; Yeates, 2014b, 2014d). Still, there remain significant gaps in our understanding of how regional social policies are playing out beyond stated orientations and objectives enshrined in regional organisations’ constitutions, laws and official documentation. In this, the papers in this collection make progress by adding to our evidence base about how specific regional organisations are progressing social policy in practice as elements of their integration strategies and processes.

The focused emphasis on health opens a window onto a broader picture of the extent to which regional organisations are not just developing regional social policies per se but onto the kinds of social policies that they are prompting. The extent to which regional organisations are promoting the values of social inclusivity and equality through regional health policies and how these are being resourced – institutionally, politically, financially – can reveal much about the political textures of regional social policy and of the extent of actual commitment to the values those organisations publicly champion. Health provides a necessary sector-specific lens through which to examine how Southern regional formations are engaging in power constellations, institutions, processes, interests and ideological positions affecting health and social welfare at different levels of governance. Thus, this Special Issue provides evidence about the ways in which regional formations are platforms on which the contested politics of globalisation and health are fought out.

The remainder of this introductory article is organised around three principal sections. The next substantive section reviews literatures spanning global social policy, regional studies and diplomacy studies, and the state of knowledge and understanding of the ‘place’ of regional actors in health governance as a global political practice. Our literature review identifies theoretical and thematic points of connection between disparate literatures and how these can be bridged through research focusing on the social policies of regional organisations and regional integration processes. The following section identifies and locates the contributions of each of the research articles to this research effort and draws out their implications therein. The final section turns to the question of the place of regionalism within global social policy as a political practice. It sets out key debates around regionalism in the context of the post-2015 development agenda and the Sustainable Development Goals and serves to introduce the *Forum* contributions and locate them within those debates.

## Locating and debating regionalism

### Regionalising Global Social Policy

Despite a wide array of political-economic projects of varying compositions, capabilities and aspirations, expectations of what regional governance can deliver have been evaluated primarily in terms of trade liberalisation and trade integration. However, as the importance of regions and regionalism increases in global politics, and integration ambitions and initiatives extend beyond trade and investment to embrace a far wider conception of social policy, there are new opportunities to explore socio-political and socio-institutional dimensions of world-regional orders in support of rights-based approaches to social development and inclusion at different levels of governance. There are now a seemingly eclectic range of theoretical literatures and analytical constructs that identify the place of regionalisation processes and regionalism within the diverse and changing landscapes of actors, institutions and policy complexes impacting human welfare and socio-economic security. In particular, Global Social Policy, Regional studies and Diplomacy Studies, each of them multidisciplinary fields of academic research, have made most contributions to on-going scholarly debates about regional social policy.

The focus of Global Social Policy as a field of academic study and research has unfolded around institutionalised efforts of global social redistribution, global social regulation and global social provision shaping national social policy and/or empowering social policy actors, individually or collectively (Deacon, 2007; Deacon et al., 2010; Yeates, 2014a). In many respects, this debate has been focused on intergovernmental, multilateral institutions such as the World Bank, I*nternational Monetary Fund* (IMF), regional development banks; the United Nations and its specialised agencies; and international non-state actors and philanthropies financing and supporting the implementation of social development goals and programmes (Yeates, 2014a). The political forces involved in global social policy formation and the arenas through which it is played out, however, are much broader than those being focused on by Deacon, while Global Social Policy is vulnerable to claims that it is decontextualised from time (history) and place (geography; Yeates, 2014c). Yeates reminds us of the *plurality of actors* in the making of global social policy, in particular the role of non-state actors and non-elites in global social politics and policy-making, notably social movement and non-governmental organisations ‘operating in the numerous shadow congresses and social fora that accompany international governmental meetings’ (Yeates, 1999: 386), and of the *plurality of sites of engagement and governance* (Yeates, 2002), including sub-global spheres of cross-border governance (Yeates, 2014c, 2014d). There is indeed a highly active set of political forces and policy actors battling over ideas and alternative policy that tend to be omitted from explanations of national policy change as a consequence of global social policy. In this context, there is an increasing recognition of the importance of regional organisations and their social policy agendas (Yeates, 2014b, 2014c; Yeates and Deacon, 2010; Riggirozzi, 2014; also Kaasch and Stubbs, 2014; Cavaleri 2014; Bianculli and Hoffman, forthcoming).

The ‘regional turn’ in global social policy is, as Yeates (2014d) argues, ‘in part a reflection of the “realpolitik” of global social policy-making and change in which there is greater acceptance in principle of the distinctive benefits of strengthened regional governance of social policy’. Yeates and Deacon (2010) and Yeates (2014b) set out several principled advantages in building a social policy dimension to regional groupings of nations, as follows:

- Regional organisations offer their member access to broader social policy options attuned to their specific contexts. Because they consist of fewer countries with more similar cultural, legal and political characteristics, they offer greater ease and pace of agreeing on common social policies, including greater possibilities for advancing their own regionally defined social standards. More developed countries can force social standards upwards in the poorer members, while smaller countries can have a strong blocking effect on the liberalising ambitions of larger ones (or vice versa).
- Regional formations offer countries enhanced access to and influence over global policy. Countries acting through regional associations can have a louder voice in global arenas instead of acting alone. Regionally coordinated responses can overcome the limitations of small-scale initiatives and are more likely to sustain the interest of prospective partners outside the region.
- Regional strategies can protect, promote and reshape a regional division of labour, trade and production to promote cooperation and generate fiscal resources for social policy. Too often global trade comes with tax exemptions for local and global companies in ways that erode domestic fiscal capacity and resources. Common regional trade and tax rules can help support and build fiscal capacity in the region that can be used to support regional social policy priorities.
- Regional social policy can enable economies of scale and the pooling of risks and resources among member countries. Regional action plans, regulatory frameworks and partnerships can address intra-regional imbalances in education, health and welfare provision and capacity. Limitations of small-scale social insurance schemes can be addressed by pooling and spreading risks regionally. Regional coordination offers the possibility of more effective preparedness for and response to disasters and other calls on aid.
- Regional groupings can provide donors and partners with a single point of contact for discussions relating to member countries. Regional groupings provide a channel through which to disburse development aid (Yeates, 2014b).

Despite this rich debate, there is still little actual empirical research into how regional organisations are actually participating in the definition and implementation of transnational social policies. Research on the regional integration–poverty nexus funded by the World Bank (Schiff and Winters, 2003) and the Department for International Development (DfID; te Velde et al., 2006) focused on the liberalisation of foreign trade, foreign direct investment and labour migration, and recognised the importance of active regional public policies in ensuring a fair distribution of benefits from economic integration. Yet, too little is known about whether poverty reduction agendas and goals are in practice being progressed through regional cooperation, and if so, how regional social policies work in practice addressing global asymmetries and across societies. This omission is not academic neglect but rather a consequence of how social concerns unfolded in regional entities as their mandates have historically focused more on trade liberalisation or (traditional) security issues. In this Issue, we take upon this task to explore how social issues are framed and manifested in regional formations. The articles by Herrero/Tussie and Penfold/Fourie, focusing on South America and Southern Africa, respectively, confirm Yeates’ (2014b) argument that regional social policies tend to have progressed faster as exhortative declarations of aims and principles rather than as binding regulatory or redistributive mechanisms. Nonetheless, the symbolic and practical uses of exhortative policy in each region indicate that political and social domains are defining region building and trajectories in the definition, pursuit and delivery of social goals. Of course, the ways Union of South American Nations (UNASUR) and Southern African Development Community (SADC) embed social mandates and commitments in their forms of cooperation and governance, as the articles indicate, are context and political contingent. Rodríguez and De Lombaerde remind us of external factors affecting regional capacity to advance and enforce health policies and rights. Nonetheless, as Amaya et al. demonstrate in their article covering several regional organisations, new and different normative frameworks are driving socio-political practices defining regional health governance and policy.

### Regions and regionalism as spaces for policy-making and action

Two and a half decades ago, debates in the political economy of regionalism and development were dominated by debt crisis, austerity and a new economy of the market – often linked to a high point of US influence over regional politics across the South (Gamble and Payne, 1996: 251–252; Phillips, 2004). Regional politics beyond the European Union (EU) especially was seen to be nested in and modelled by demands for trade and financial deregulation, or the creation of security communities, captured by ideological and political constrictions, securing at the same time regional spheres of influence (Buzan and Wæver, 2003; Hirst, 2003; Nel and Nolte, 2010). Regionalism was seen as manifestations of global orders, envisioned as hegemonic politics triggered by the need to engage efficiently in global market activity. Regionalism from this perspective was conceived as a building block to global liberalisation through the interplay between state-led macro-processes of regulation and micro, and often informal, processes of regionalisation led by business and other non-state actors. This persuasive argument proved resistant to many claims about historical roots supporting different pathways to regionalism (Mansfield and Solingen, 2010) – diverse dynamics of cooperation in different areas of regional policy (Gomez-Mera, 2008; Riggirozzi and Tussie, 2012) and across regional geographies (Söderbaum and Van Langenhove, 2006). The assumption is not surprising as policy makers and social groups from the developed world have powerful resources to set agendas and act as rule-makers in global politics, and in many cases is legitimate in many counts of international policy-making and international relations. At the same time, it is not comprehensive; indeed, it neglects significant trends – some established, others emergent. To an extent, similar dilemmas face global policy makers today, but the context is different.

Given the losing grounds and even legitimacy of neoliberalism as a political-economic project structuring Southern national and regional governance, and given the changing coordinates of geo-political interests and power in recent years, there is a renewal of development strategies that are played out differently in different policy spaces, particularly in Southern world regions. Since the late 1990s, regional organisations have started to tackle questions of the relationship between trade, labour and social standards, and of how to maintain fiscal capacity and social solidarity in the face of international competition (Yeates and Deacon, 2010). In light of this, this Issue explores how regional groupings of countries are embracing new agendas and developing plans of action to achieve social development through regional health policies and governance. This is particularly significant in regions where poverty is a driving force of under-development and social inequities. Although neglected partners in global efforts tackle poverty, regional organisations offer distinctive opportunities to strengthen institutional actions on poverty and equity.

The time to review these ambitions and how they manifest in regional organisations in the South is propitious because political action on poverty and health inequalities has emerged as a key area driving regional politics in the South addressing the most vulnerable through justice and equity claims in a normative sense, as ‘right to development’ (Grugel and Piper, 2009) or ‘human right to health’ (Hayden, 2012), based on the recognition of state obligations to create opportunities and capabilities to enjoy those rights, and echoing growing global awareness of the interplay between rights and social development with the signature of the Millennium Declaration, the Oslo Declaration and more recently the Sustainable Development Goals, there was a renewed focus on the links between global poverty and human rights in development (*The Lancet*-University of Oslo Commission on Global Governance for Health, 2014).

### Regionalising global health through diplomacy

Another way of assessing the increasing role of regional organisations and the synergies with action on poverty reduction relates to the capacity of regional organisations to act as a bloc, broker or actor in global governance through new modalities of ‘regional diplomacy’ (Riggirozzi, 2015). We argue that Southern regional organisations can play a significant role in health diplomacy, variously creating normative frameworks or acting as a bloc in the advocacy of, and acting as, alternative to established structures and norms of global (health) governance. This argument contributes to existing debates about health diplomacy and diplomacy studies which have tended to focus on individual governments as agents of policy and policy diffusion (Buss and do Carmo Leal 2009; Kickbusch and Ivanova 2013). Diplomacy is an expanding area of study and practice which although established in International Relations is now also taking hold in Public Health. Trans-border efforts of collective action cooperation in health, but only, are not new and can be traced back to the 19th century when negotiations on health were linked to surveillance and control of certain communicable diseases such as yellow fever, cholera and plagues. These efforts of health diplomacy (or activism) were oriented to improve responses to transnational problems and reduce the burden on national health systems, as well as to keep healthy corridors of trade (see Herrero and Tussie and Cooper and Farooq in this Issue). The beginning of the 20th century certainly expanded collective action towards new health-related issues, such as mitigation of pollution in rivers and lakes, regulations in the trade of alcohol and tobacco, the obligation to treat wounded soldiers during war, and treaties to protect workers’ safety and health in their occupational environment (Fidler, 2001). The creation of the World Health Organization (WHO) in 1948 has been a milestone for global health diplomacy, institutionalising sanitary regulations and norms for the control of infectious diseases and establishing a regulatory framework for global health governance (Fidler, 2001: 843). Caught in the geo-political competition during the Cold War, however, it was not until the mid-1960s onwards when the WHO found significant political and intellectual impetus from state actors and social movements concerned with the human and social aspects of development which stimulated further thinking about development cooperation (Fidler, 2011). But the real ‘revolution’, according to Fidler (2004: 45), manifested in the 21st century as health emerged from a neglected position in international politics to a prominent place in the global agenda as the world faced the crises posed by infectious diseases, normative upheaval in tackling these crises and in many cases the lack of authoritative power of the global institutions to mobilise material resources and technical capabilities to contain and mitigate the threat (Fidler, 2004: 2). We are interested in how regional organisations are placed in this revolution and what sort of insights they bring as well as to the dynamics they create in health governance.

The literature on global health diplomacy and governance, which has developed rapidly over recent years, focuses on either around the governance roles of specific international multilateral institutions (e.g. WHO, World Bank, United Nations Children’s Fund [UNICEF], Joint United Nations Programme on HIV/AIDS [UNAIDS]), non-governmental organisations (e.g. Médicins Sans Frontieres, Oxfam, the Gates Foundations) and public–private partnerships (e.g. the Vaccine Alliance -GAVI), or the governance of particular health problems, most commonly infectious diseases (Rushton and Williams, 2011). This is not surprising as policy makers and social groups from the developed world have powerful resources to set agendas and act as rule-makers in global politics. Harman (2010), for instance, argues that the highly centralised nature of decision-making and delivery in global governance, led by a state-centric and hierarchical mode of organisation, has the effect of ‘pigeon-holing issues and prescribing interventions’ while reproducing a power gap between international institutions and donors (i.e. the World Bank, the Bill and Melinda Gates Foundation, states within the G8) and the governments and civil society actors within developing countries. In other words, who frames what and why depends on how actors are positioned and negotiate interests in global governance and health. Dominant (international) frameworks risk pushing for universal norms to (social) development that in practice can be turn either conservative or a-contextual. William Easterly (2009) claims that ‘*which* rights to health are realised is a political battle’ contingent to a political and economic reality that profits on the margins of (poor) health. For instance, diseases like HIV, malaria and tuberculosis account for over 90% of the global disease load, while the other more ‘neglected diseases’ like dengue, Chagas and other tropical diseases affect disproportionally societies in the South. Yet HIV/AIDS accounted for 57% of World Bank projects on communicable diseases from 1997 to 2006, compared with 3% for malaria and 2% for tuberculosis. *Other big* killers of the poor – such as pneumonia, measles and diarrheal diseases, which together accounted for more than 5 millions of deaths in 2008 – also received little attention.

In other words, claiming, framing and advancing norms and policies have been associated with the global power of Northern actors. Less is known about the place of regional organisations in global health diplomacy. Some empirical work on the role of Southern actors in support of health cooperation has been produced (see Bliss, 2010; Kickbusch and Ivanova, 2013; Onzivu, 2012). However, current scholarly writings tend to emphasise diplomatic interactions led by singular ‘regional powers’, primarily Brazil, South Africa or China, in international diplomacy and within the WHO. While important global players in health diplomacy, the challenge is less one of depicting how member states’ interests play out in the global system or the role of NGOs – mainly operating from major Western countries, but how to explain how regional organisations can become political structures that provide effective opportunities and incentives to undertake collective action, and fundamentally how a regional polity can *itself* become a policy entrepreneur brokering demands and reworking (global) rights to health.

Riggirozzi (2015: 414) has taken up this point to argue that regional organisations can act as ‘corrective devices and moral vectors in global health governance’ engaging in specific modalities of mobilisation and offering diplomatic and strategic options to advocacy actors. As such, the argument goes, regional organisations and identities must be considered important keywords in advocacy and contention politics, as well as in the academic analysis of who acts, who frames and who contests global (health) policies. She goes on to claim that, as states pool resources together, they can play a distinctive role by (1) providing regional normative frameworks that can structure practices in support of social policies and rights-based governance, (2) creating opportunities for (re)allocation of material and non-material resources and thus for inclusion and (3) acting as unified representative actors in global political space enabling representation and claims-making, contesting and reworking global governance in support of global justice goals (Riggirozzi, 2015: 416). In different ways, the research papers and The Forum substantiate these arguments and point to the need to investigate the relations of the regional level of analysis *between* the state and the globe, and the processes that connect regional and national politics *within* the regional space if we are adequately to analyse contemporary forms of power, regional activism and cooperation on health and other social issue areas. Poverty-focused social policy research into what regional associations are doing as well as what they are saying is needed to help all stakeholders, locally, nationally and internationally to know what they can do to support pro-poor policies and programmes of concerted social action. Regional organisations can provide platforms for practitioners, academics and policy makers to collaborate and network towards this aim. Changes in global (health) diplomacy are taking place and warrant deeper study of the place of regional organisations in advancing and contesting new norms of governance. Neglecting this jeopardises effective development aid and the global agenda related to the sustainable development goals.

## Overview of articles in this Special Issue

The papers in this issue are taken from the above discussion. Amaya, Kingah and Rollet first explore how Association of Southeast Asian Nations (ASEAN), EU, SADC and UNASUR frame health as a foreign policy issue and how this has an impact on their prioritisation of policies. The policy frames identified they argue respond to challenges these regions face. For instance, ASEAN’s struggle with re-emerging diseases has led to favouring a securitisation approach to health, the EU approaches health as a cross-cutting policy issue, SADC presents health as a driver for development, while UNASUR emphasises addressing social determinants of health as an ethical imperative. This is an important baseline analysis as framing, it could be argued, potentially supports not only different institutional frameworks but also very different kinds of collective action and allow different sorts of claims to be made by groups and movements, transnational activist networks, epistemic communities or policy entrepreneurs (Keck and Sikkink, 1998; Risse et al., 2009, 2013). While institutional settings and political regimes shape the opportunities and demands upon which actors are mobilised (Tarrow, 2001), the impact of regional organisations according to Amaya, Kingah and Rollet depends on their ability to harness their convening power and speak in a coherent voice on health matters.

This analysis is followed by Herrero and Tussie’s focus on UNASUR. Established in 2008, it has had from the outset a clear emphasis on reducing the regional social deficit. Within this mission, health policies became a strategic factor in South America to collectively balance the legacy of neoliberal policies in the region. The paper describes the social, political and economic processes that explain the emergence of UNASUR and its focus on social policy through healthcare. It is argued that by virtue of UNASUR’s Health Council, healthcare became the spearhead of cooperation giving way to novel forms of diplomacy within and outside the region.

Also with a focus on the South, the paper offered by Penfold and Fourie analyses how regional health is framed in the SADC and what type of governance arrangements and (mis)opportunities of diplomacy in relation to social protection as a mechanism to address health issues. This paper assesses the role of SADC in health governance and argues that SADC has the potential to play more of a role in facilitating health policy and access to healthcare and medicines in the region.

The last paper with an emphasis on policy-making in the South is offered by Lizarazo Rodríguez and De Lombaerde, who take a different stand in relation to health policy and diplomacy. Accordingly, while it can be argued that in the area of health policies there are important opportunities to adopt regional approaches to tackle border-crossing health issues, the article draws the attention to the fact that the linkage between international/regional and national policy levels is not unidirectional. Existing regional trade arrangements do not necessarily offer the kind of hopes for progressive regional health governance expressed in the UNASUR and SADC articles. The article explores contradictory tendencies by exploring legal provisions managing the tension between the promotion of free trade and the enforcement of the right to health in the case of Colombia.

The closing article of the Issue is dedicated to a reflective analysis by Cooper and Farooq who speculate on whether health diplomacy is re-shaping the conceptualisation and practice of diplomacy. The article contends that the increased number and diversity of actors in the global health arena have changed the conceptualisation and practice of diplomacy, and a greater plurality in health governance. Furthermore, health diplomacy, because of its special character, opens up the possibility of diplomacy being done differently in subtle ways in micro-level, bringing to attention the issues, tensions and governance processes identified in the overall Special Issue.

## Forum: Regionalism and the post-2015 development cooperation agenda

A foremost critical challenge confronting many countries is how to develop a regionalism that will underpin efforts to achieve social development objectives and accelerate the realisation of the right to health and the ending of the global public health crisis. This is an agenda which many regional organisations and networks of civil society activists are already addressing, but all too often regional formations as sites of governance and policy-making are bypassed or ignored in on-going campaigns to strengthen social provision and rights in support of health. The acute relevance of thinking and acting regionally *as well as* locally and globally when it comes to strengthening health provision becomes especially apparent in the context of current global social policy and development agendas. The definition and delivery of international development goals need effective and robust frameworks that can mobilise and sustain the necessary political and policy impetus among the widest range of actors over time. Regionalist modes of cooperation, coordination and governance are in this context a vital feature of those frameworks.

Viviene Taylor opens *Forum* with a powerful, eloquent and passionate statement of why regionalism is a key ‘missing link’ in the global and national politics and policy of social development. With particular reference to African contexts, she argues that although regional integration agendas no longer remain limited to trade and financial integration progress by regional and international communities to address how to better develop and harness more substantive regionalist institutional arrangements in support of inclusive *social* development remains lamentably slow. Setting out the challenges of developing a strengthened regional agenda on inclusive and pro-poor social development in practice, she unequivocally highlights that ‘Reconceptualising poverty eradication strategies within an African regional agenda that is part of the African Renaissance project could provide political and moral legitimacy for such social policy interventions’. This regional project and the multiple strategies it devises to enact this should be guided by the values and principles of human rights, democracy, transparent and accountable governance, active participation of citizens and civil society organisations. The necessity of a strengthened regionalism in which human rights are firmly embedded resonates strongly with the article by Dainius Puras, the *UN Special on the right of everyone to the enjoyment of the highest attainable standard of physical and mental health*. Human rights and the right to health, he argues, need to be embedded in the Sustainable Development Goals. Human rights are, then, indispensable to a sharper and more consistent focus on regional governance and inclusive social development in the post-2015 Sustainable Development Goals implementation framework.

The possibilities of Southern regional organisations having a more substantial role not just in implementing global development agendas but also framing and shaping the ideas and principles that underpin them must be taken more seriously. Mariana Faria highlights a critical feature of the work of the UNASUR, notably its efforts to advance and embed a social determinant of health perspective in the post-2015 development agenda. She also identifies key challenges for UNASUR in consolidating and extending its emerging track record as a regional actor capable of advancing progressive policy agendas focused on poverty eradication and inclusive development: gaining recognition as a bloc actor in institutions of global governance, building alliances with other regions and countries in support of its positions and values, generating data and evidence about regional priorities to underpin common positions and strengthening UNASUR’s alliance with South American civil society and academicians in support of the values it advances.

The question of how to develop a strengthened regional agenda which is not confined to implementing donor-defined and donor-funded initiatives is also discussed by Keneilwe Mooketsane and Molefe Phirinyane and by Erica Penfold in relation to Africa. Acknowledging reliance on donors in development initiatives in Africa, Mooketsane and Phirinyane argues for a discernible and more visible role for the African Union and the African regional bodies such as SADC and E*conomic Community of West African States* (ECOWAS) in leading the development of African regional health strategies involving multiple development partners drawn from African states, civil society, the private sector and donors. They highlight the responsibilities of donors in helping to bring about the conditions for this, where one crucial element is to support the regionalisation of health and inclusive social development agendas along with the institutional frameworks capable of enacting them.

Echoing this, Penfold’s article specifically identifies the responsibilities of the WHO in this regard and highlights the need for it to work more closely with the *African Union* (AU), *East African Community* (EAC), SADC and ECOWAS to support a strengthened regional health governance that prevents severe health crises rather than react to them. She also draws out more precisely the challenges in actually growing a strengthened regional health governance in the continent: unequal political power between governments, civil society and donors, and the proliferation of undemocratic governments that do not always regard civil society as a development partner. Clearly, extant regional bodies have a leadership responsibility to become more effective with the mandates they do have, and she comments critically on SADC’s failure to ensure regional oversight of vaccine procurement and immunisation programmes. She urges SADC and other actors to rethink the place of regional governance within the post-2015 SDG and implementation framework in order to ensure that health security and justice become actual outcomes rather than remain an aspiration.

Jenina Joy Chavez reviews the ‘place’ of regional governance in the global politics of a major global public health issue – tobacco and its control – drawing on key findings from her on-going research into the social impacts of trade liberalisation in the Southeast Asian context, more particularly in relation to ASEAN. She argues that despite the increased activism of ASEAN health officials on tobacco control, they are hampered by their lack of mandate on economic issues which is firmly located within the realm of trade and its liberalisation and by the lack of progress by ASEAN health and trade officials in finding ways of pursuing a public health agenda together. She urges them to do so to harmonise public health and economic objectives. Here, the role of public health activists in addressing regional economic and not just health policy in order to affect health outcomes becomes paramount: They need to be better prepared to move from broad critiques of trade liberalisation to make specific, appropriate and effective requests and demands in the interests of public health protection. Given the presence of regional organisations in global trade liberalisation and integration projects, regional agendas and organisations should move much more to the foreground of transnational public health activism. In short, public health communities need to develop their own practices of regional pro-health activism and diplomacy which include the arena defined as regional health policy but which also address other sectoral policies impacting health. The role of regional actors (including regional organisations themselves) in stimulating expansive regional agendas capable of integrating and balancing multiple agendas impacting health is clearly paramount in the realisation of people-centred inclusive social development in the post-2015 era.

There are signs that the post-2015 sustainable development round is cognisant of the potential role of regional fora in global health governance. Mami Sakurai from United Nations Office for South–South Cooperation (UNOSSC) highlights how the Secretary-General of the United Nations’ (UNSG) report on post-2015 sustainable development foresees a role for regional forums – by, for example, developing South–South technical assistance and the sharing of experiences. The potential role of multilateral development organisations and donors in providing financial support to stimulate and support such initiatives is not inconsiderable; here, regional bodies are obvious extant regional partners with mandates, institutional capacity, networks and resources to take up this leadership role post-2015. She concludes by signalling the need for further research and analysis on existing good practices and the potential of regional bodies and partnerships in order to feed into on-going discussions about the implementation of sustainable development not just as a set of abstract goals but as a living reality.
